# Beyond guideline knowledge: a theory-based qualitative study of low-value preoperative testing

**DOI:** 10.1186/s13741-023-00292-5

**Published:** 2023-03-02

**Authors:** Yamile Jasaui, Sameh Mortazhejri, Shawn Dowling, D’Arcy Duquette, Geralyn L’Heureux, Stefanie Linklater, Kelly J. Mrklas, Gloria Wilkinson, Sanjay Beesoon, Andrea M. Patey, Shannon M. Ruzycki, Jeremy M. Grimshaw, on behalf of the De-Implementing Wisely Research Group

**Affiliations:** 1 Continuing Medical Education, Cumming School of Medicine, University of Calgary, Alberta, Canada; 2 Centre for Implementation Research, Ottawa Hospital Research Institute, Ottawa, ON Canada; 3 School of Epidemiology and Public Health, University of Ottawa, Ottawa, ON Canada; 4 Department of Emergency Medicine, Cumming School of Medicine, University of Calgary, Alberta, Canada; 5 Patient Partner, De-Implementing Wisely Research Group, Edmonton, Canada; 6 Strategic Clinical Networks, Provincial Clinical Excellence, Alberta Health Services, Edmonton, AB Canada; 7 Surgery Strategic Clinical Network, Alberta Health Services, Edmonton, AB Canada; 8 Faculty of Medicine and Dentistry, University of Alberta, Edmonton, AB Canada; 9 Department of Medicine, Cumming School of Medicine, University of Calgary, Alberta, Canada; 10 Department of Medicine, University of Ottawa, Ottawa, ON Canada

**Keywords:** Preoperative testing, Electrocardiograms, Chest X-rays, Anesthesia management, Theoretical Domains Framework

## Abstract

**Background:**

Choosing Wisely Canada and most major anesthesia and preoperative guidelines recommend against obtaining preoperative tests before low-risk procedures. However, these recommendations alone have not reduced low-value test ordering. In this study, the theoretical domains framework (TDF) was used to understand the drivers of preoperative electrocardiogram (ECG) and chest X-ray (CXR) ordering for patients undergoing low-risk surgery (‘low-value preoperative testing’) among anesthesiologists, internal medicine specialists, nurses, and surgeons.

**Methods:**

Using snowball sampling, preoperative clinicians working in a single health system in Canada were recruited for semi-structured interviews about low-value preoperative testing. The interview guide was developed using the TDF to identify the factors that influence preoperative ECG and CXR ordering. Interview content was deductively coded using TDF domains and specific beliefs were identified by grouping similar utterances. Domain relevance was established based on belief statement frequency, presence of conflicting beliefs, and perceived influence over preoperative test ordering practices.

**Results:**

Sixteen clinicians (7 anesthesiologists, 4 internists, 1 nurse, and 4 surgeons) participated. Eight of the 12 TDF domains were identified as the drivers of preoperative test ordering. While most participants agreed that the guidelines were helpful, they also expressed distrust in the evidence behind them (knowledge). Both a lack of clarity about the responsibilities of the specialties involved in the preoperative process and the ease by which any clinician could order, but not cancel tests, were drivers of low-value preoperative test ordering (social/professional role and identity, social influences, belief about capabilities). Additionally, low-value tests could also be ordered by nurses or the surgeon and may be completed before the anesthesia or internal medicine preoperative assessment appointment (environmental context and resources, beliefs about capabilities). Finally, while participants agreed that they did not intend to routinely order low-value tests and understood that these would not benefit patient outcomes, they also reported ordering tests to prevent surgery cancellations and problems during surgery (motivation and goals, beliefs about consequences, social influences).

**Conclusions:**

We identified key factors that anesthesiologists, internists, nurses, and surgeons believe influence preoperative test ordering for patients undergoing low-risk surgeries. These beliefs highlight the need to shift away from knowledge-based interventions and focus instead on understanding local drivers of behaviour and target change at the individual, team, and institutional levels.

**Supplementary Information:**

The online version contains supplementary material available at 10.1186/s13741-023-00292-5.

## Background

Preoperative testing before low-risk surgeries, referring to procedures that have a baseline risk of adverse events of less than 1% (Kirkham et al. 2015), does not improve patient outcomes (Chan et al. 2011; Biteker et al. 2012; Fritsch et al. 2012; Smetana and Macpherson 2003), increases costs to the health care system, (Rayborn et al. 2017; Finegan et al. 2005; Kash et al. 2015), and can prolong time-to-surgery and length of stay (Bernstein et al. 2016). Based on extensive evidence, Choosing Wisely Canada and most major anesthesia and preoperative guidelines recommend against obtaining any preoperative tests before low-risk procedures (Dobson et al. 2021; Canadian Anesthesiologists’ Society Five Things Clinicians and Patients Should Question n.d.). However, while research shows that guidelines may have modest to moderate improvements in care, they alone do not reduce low-value test ordering (Grimshaw et al. 2004). For example, in 2012–2013, 17.9% of patients undergoing low-risk procedures in a single Canadian healthcare system had at least one low-value electrocardiogram (ECG) or chest X-ray (CXR) (Canadian Institute for Health Information 2017), compared to 18.9% in 2015–2016, 14% in 2017–2018, and 11.7% in 2019–2020 (Canadian Institute for Health Information 2022).

Designing interventions to reduce low-value preoperative testing (defined as an ECG or CXR performed in a patient undergoing a low-risk surgery) requires understanding the current drivers of low-value test ordering by clinicians (Health Professions Networks Nursing & Midwifery for Human Resources of Health (2010); Kvarnström 2009). Previous theory-informed study of preoperative test ordering behaviours in anesthesiologists and surgeons in Ontario, Canada by Patey et al. (2012) found that low-value test ordering is due to systems- and individual-level factors. These factors include lack of clarity about which healthcare provider was responsible for ordering preoperative tests, a “just-in-case” mentality of ordering investigations to prevent adverse patient outcomes and surgical cancellations, and low-value tests being completed before the anesthesiologist appointment (Patey et al. 2012). However, it is not known how these drivers differ between healthcare systems. Building on this work, we sought to understand drivers of low-value preoperative testing among anesthesiologists, internal medicine specialists, nurses, and surgeons in a single healthcare system in Alberta, Canada. The aim of this work is to (1) explore how context (i.e., health systems in different jurisdictions) influences identified barriers and (2) match these drivers to targeted interventions to reduce unnecessary testing in surgical patients undergoing preoperative assessment.

## Methods

This qualitative study used theory-guided, semi-structured interviews to examine drivers of low-value preoperative test ordering behaviours among preoperative healthcare providers. For the purposes of the study, low-value preoperative tests were defined as ECGs or CXRs in patients having low-risk surgeries (Kirkham et al. 2015) in keeping with previous studies by study team members. Low-risk surgeries are non-urgent procedures with a less than 1% risk of cardiac death or myocardial infarction; examples include endoscopies, ophthalmic procedures, and arthroscopy (Kirkham et al. 2015). This study was approved by University of Calgary Conjoint Health Research Ethics Board (REB18-1097) and is reported according to the Consolidated criteria for Reporting Qualitative Research (COREQ) guidelines (Tong et al. 2007).

### Setting

Alberta is a Canadian province of over 4.5 million people with public health insurance under a single healthcare system. Patients undergoing a scheduled surgery may be referred for a preoperative medical or anesthetic consultation by the operating surgeon depending on local guidelines or individual assessment of the patient’s medical comorbidities. Depending on local resources, patients may see an internist, family physician, anesthesiologist, or some combination of these providers in preoperative consultation.

### Participants

Healthcare providers involved in preoperative test ordering (anesthesiologists, internists, nurses, and/or surgeons) were recruited using a snowball sampling strategy (Brewerton and Millward 2012) and purposive sampling techniques (Maxwell 1996). First, we asked clinician-researchers on the study team to identify 2–3 providers who might be knowledgeable about preoperative test ordering. Then, as part of the interview script, we asked consenting participants to suggest potential clinician participants who may have differing opinions for participation.

Inclusion criteria were physicians or nurses currently practicing preoperative assessment, care, or management, and who had seen patients in this capacity in the previous 3 months in Alberta, Canada. An environmental scan prior to this project suggested that ordering preoperative tests was in the scope of practice for nurses working at certain sites and that some nurses were ordering preoperative tests on behalf of other healthcare providers outside of their scope of practice. For this reason, we included nursing in our recruitment to avoid missing data on drivers of low-value preoperative testing. We purposively sampled to ensure we had participants from rural and non-academic settings and varying years of clinical work experience. Considering the need to sample four different clinician groups in rural and urban areas, we estimated a sample population of 25 participants, with at least 5 per discipline (French et al. 2012). We reviewed the demographics of the sample after 10 interviews and focused recruitment on underrepresented disciplines. We used the 10 + 3 data saturation approach (Patton 2002); in this approach, we began analysis of themes after an initial sample of 10 participants and continue recruitment until 3 consecutive interviews did not contribute new themes. Saturation was reached after 19 interviews.

### Interviews

A semi-structured interview guide was developed using the Theoretical Domains Framework (TDF; version (1), a theory that guides study of the drivers and barriers to a behaviour (Michie et al. 2005), and previous work by study team members (Patey et al. 2012) (Additional file 1). The final interview guide consisted of 35 questions, including questions about their workplace and questions based on each of the 12 domains in the TDF: *knowledge; skills; social/professional role and identity; beliefs about capabilities; beliefs about consequences; motivation and goals; memory, attention and decision processes; environmental context and resources; social influences; emotion;* and *behavioural regulation*. At the beginning of the interview, participants were provided with operational definitions of low-risk surgery and low-value preoperative testing to ensure consistency across responses. The interview guide was piloted with a clinical team member (SMR) and did not require revision. Potential participants were contacted via e-mail and invited to participate in an interview. Interviews were conducted via telephone or in-person.

### Data analysis

Interviews were audio-recorded, de-identified, transcribed verbatim, and loaded into NVivo 12 (Burlington, MA) for coding and analysis. A coding guideline based on previous research (Patey et al. 2012) was used as an initial guide. Two team members (YJ and SM) applied the deductive coding framework to the first transcript and reconciled discrepancies through discussion. Investigators then independently coded interviews in NVivo using Framework analysis (French et al. 2012). Cohen ‘s Kappa coefficient (Landis and Koch 1977) was used to determine agreement between reviewers for all assigned codes. Domains with Cohen ‘s Kappa coefficient < 0.80 were discussed between research team members to reconcile discrepancies. If consensus could not be reached, discrepancies were referred to the team’s health psychologist for resolution (Landis and Koch 1977). Relevant domains were identified by one researcher (YJ) and confirmed by a health psychologist (AMP) based on (1) how many times a belief appeared across interviews, (2) presence of conflicting beliefs, and (3) the perception of how strongly a belief influenced the behavior (Patey et al. 2012).

After coding, one researcher (YJ) reviewed all utterances and wrote a summary sentence which captured the key message or specific belief expressed in each. Similar key messages by the same participant were only reported once. Belief statements were then grouped based on similarity to create themes and reviewed by the health psychologist (AMP). A table consisting of the major themes, specific beliefs, and participant quotes was created for each of the domains with a final column indicating the number of participants sharing each belief.

## Results

### Participant and site demographics

Participants were diverse with respect to practice discipline, academic appointment, and geographic location of practice (Table 1). Data from three nurse participants was not included in this analysis as they reported from the outset that they were unable to order preoperative tests. This did not change data saturation.

**Table 1:** Demographics and work characteristics of interview participants

Participants	Anesthesiologists (*n* = 7)	Internists (*n* = 4)	Surgeons (*n* = 4)	Nurses (*n* = 1)
Sex				
Male	3	4	3	–
Female	4	0	1	1
Setting				
Academic	4	2	4	–
Community	2	1	0	1
Academic + community	1	1	0	0
Clinical experience (years; median (IQR))	11.0 (1–18)	15.5 (5–20)	13.0 (3–33)	–
Proportion of their patients who undergo low-risk surgeries^a^	30–100%	20–50%	10–50%	100%

^a^Participant estimates

### Key themes

In total, 1,852 utterances from the 16 interviews were coded into the 12 TDF domains. Interrater reliability for each interview ranged from ‘substantial agreement’ (*k* = 0.67) to ‘almost perfect’ (*k* = 0.87; mean 0.77 ± 0.07) (Landis and Koch 1977). Key themes from the interviews were found across eight of the 12 theoretical domains: *knowledge, social/professional role and identity, beliefs about capabilities, beliefs about consequences, motivation and goals, environmental context and resources, and social influences* (Table 2).

**Table 2:** Examples of belief statements and sample quotes from anesthesiologists (A), internists (I), surgeons (S), and nurse participants (N) assigned to the theoretical domains identified as relevant

Domains	Specific belief	Sample quote	Frequency out of 16
Knowledge	Guidelines are helpful	“I think that they should definitely exist. They are really important to reduce unnecessary testing, and to standardize patient care pre-operatively. I think that there should be a solid guideline for all the pre-op testing that we do.” (A2)	10
	There is evidence to support the guidelines	“From what I’ve seen for the most part, yes. They seem to be…” (A7)	8
	I do not know all of the evidence in guidelines	“[…] I’m not aware of who developed the guidelines or what the evidence behind them is…” (S1)	5
	The quality of the evidence is not good	“…So far, most guidelines are based on a few small studies. My interpretation of—is that we would—it—we need stronger evidence.” (A1)	3
	Quality of evidence behind guidelines varies	“[…] The evidence is actually very contradictory, because there’s different—it really depends on the data set that you use. There’s many guidelines that are based on local situations that just don’t exist in Calgary.” (I1)	2
Social/professional role and identity	I (internist/anesthesiologist/surgeon) should be responsible for ordering ECGs/CXRs	Realistically, it better be the surgeons [responsible for ordering ECGs/CXRs], because a low-risk patient shouldn’t have to go through a pre-admission clinic.” (S1)	9
		“Either anesthesiologists or internal medicine. […] I’d say mostly anesthesiologists, because they understand better what type of procedure and what anesthetic is involved.” (A2)	
	The roles and responsibilities of the different specialties for pre-op assessment are not clearly defined	“…I think it’s more the system. I think we have a system in place where a patient is first seen by a surgeon, and then maybe by a nurse in the pre-admission clinic who has a clinical practice and a jurisdiction that allows them to order certain investigations, or maybe by an anesthetist in the pre-admission clinic, or any caregiver along the way.” (I2)	5
		“Sometimes the surgeon will order tests because, just because without any reason for ordering, and sometimes internal medicine will order.” (A2)	
	I sometimes do not play a role in the ordering of tests—other HCPs do	“In our clinical environment, it’s the surgeons that order the chest x-rays. We [internal medicine] don’t, and anaesthesia doesn’t.” (I1)	5
		“but it also depends, because sometimes internal medicine sees the patient, and then there is—individual physicians will order tests following their own criteria.” (A2)	
	I order tests to justify the consultation	“ECGs, I must admit I typically order them, usually because I’m wondering why I’m even seeing the patient. I’m going, “There must be something going on here that I am missing. “Right?” (I1)	2
Beliefs about capabilities	It is easy for me to not order/cancel tests	“If there’s—for example, if we’re using a sunrise clinical manager, and there’s an order set and I see that something’s been ordered that doesn’t need to be, then I’m happy to cancel that.” (A3)	13
		“If I don’t need to order anything, I don’t need to order anything, so it’s easy.” (S2)	
	I am confident that I can perform a pre-op evaluation without testing	“Yes… [I am confident to perform a pre-op evaluation for a low-risk surgery without pre-op tests].” (I2)	12
	It is too easy to order tests	“I guess the problem is that it’s so easy for me to do an ECG that I have to have a really good reason not to order one.” (I1)	10
	I am comfortable proceeding without testing	“Yes, for most surgeries [I am comfortable proceeding without testing]. Again, it depends on if it’s low risk with a low-risk patient, yes.” (S1)	9
	It is difficult for me to cancel tests ordered by another physician	“I don’t routinely in my low-risk surgeries order the tests, but if—let’s say the surgeon orders it, I can’t cancel the surgeons order.” (A6)	6
		“Even if I were to contact a referring physician and to say there’s no need to do a chest x-ray in this particular case, that wouldn’t necessarily prevent a chest x-ray from being done.” (I2)	
Beliefs about consequences	ECGs/CXRs are unnecessary for patients undergoing low-risk procedures	“Tests are unnecessary and are not going to identify [conditions] that would either be treatable or exist or influence treatments like surgery." (S1)	10
		“…those tests are of no value in the patients where they’re not indicated.” (A4)	
	Reducing unnecessary tests would save time, money, and resources	“…We need to be judicious in ordering our investigations, because we need to be responsible to the limited resources that we work within." (I2)	10
	Tests are ordered to prevent surgery cancellation or post op complications	“My main purpose in that setting is to prevent a cancellation of the surgery. So, if I anticipate that… there is a reason that the anesthesiologist of the day will cancel their surgery based on the absence of some sort of information, then I will order the tests so they have that available.” (I3)	7
	Tests are ordered to prevent problems in the OR	“I order them for myself, so I don’t have any problems in the operating room.” (S2)	4
		“We’ll just go ahead and get an ECG, because it’s, I think, still—it’s safer to have one onboard that everybody can look at, then to ignore it, and then something does happen.” (N1)	
	Ordering routine tests may catch something that’s asymptomatic	“…The negative could be if I don’t order a chest x-ray and the patient comes in in six months’ time with lung cancer, which could have been detected, or a heart attack on the ECG which could have been detected if I had done the EKG, so that is a negative.” (I4)	3
Motivation and goals	Ordering pre-op tests is sometimes important to me	"It’s important if it’s clinically necessary, and if it’s not clinically necessary, it’s not important at all.” (A5)	9
	Ordering pre-op tests isn’t important to me	“To do it, I don’t think it’s necessary to do as part of the pre-operative workup. I don’t think it’s an incomplete pre-op workup if you don’t do it.” (S1)	7
Environmental context and resources	My environment (access to ECGs/CXRs) makes it easy to order tests	“I see these patients in an environment where I can order the tests and get them pretty much immediately. There’s no environmental restriction…” (I1)	10
		“Because we have nurses that will put in all of our orders for us, and all the requisition sheets are available as well as our computers and the centre’s clinical managers. Whenever I’m logged into a patient, it’s fairly easy to order anything I might need for that patient.” (A3)	
	Hospital/departmental guidelines/policy dictates whether tests are ordered/not ordered	“Because we have a policy within the department. So most people just follow the department policy.” (A1)	7
	Due to clinic setup for efficiency, tests are ordered before I see the patient	“It’s easier if everybody has an ECG because if you have to wait until you could see the patient… it disrupts the flow of the patients in a busy clinic because the high pre-op clinics are high volume, so if the ECG is automatically done in rotation on everyone’s chart, then that makes the flow of the clinic faster.” (I3)	4
Social influences	My colleagues and I generally have similar opinions about test ordering	"Yeah [colleagues generally agree on pre-op testing practices], because we have discussion every couple of years. We discuss as a group considering our own culture, our own surgical culture, our own patient populations. Our experience with certain surgeons, so yeah, we as a group. Generally, I mean, there’s always going to be outliers but, for the most part, we reflect together every few years." (A1)	15
		"I would like to say yes, because, again, we do meet on a regular basis, and we discuss these things.” (I1)	
	The views/opinions of others don’t/rarely influence my decision to order/not order tests	“Other’s opinions may not matter if I have done my job properly because that’s why I’m here for my patients. If I’m practicing evidence-based medicine, then I should not be influenced by the opinions of others.” (I4)	8
		“I wouldn’t expect a surgeon to make decisions that would change my management. I would not expect. Yeah. For example, I wouldn’t expect a surgeon to say, "Oh, this patient needs a chest x-ray because they might have a general anesthetic." I wouldn’t expect them to know what would change my practice and what wouldn’t.” (A3)	
	I would only order pre-op tests if a physician requests them	“So, if I anticipate that if there—based on the history and physical—that there is a reason that the anesthesiologist of the day will cancel their surgery based on the absence of some sort of information, then I will order the tests so they have that available.” (I3)	4
	Other HCPs (nurses, surgeons) are ordering tests and they shouldn’t	“…as a result, some patient’s getting lab tests or that, you know, if a physician had seen them first, they wouldn’t be ordered because it’s being based on a nursing history not a physician assessment.” (A1)	3
		“Yes, I would [prefer surgeons to order pre-op tests] if they could do it appropriately, I think that would be nice, but because they can’t, the grid is fantastic for that.” (N1)	
	The views/opinions of others might influence my decision to order pre-op tests if not ordering could lead to negative outcomes (surgery cancelation, negative patient outcomes)	“Sure, it would be done [view or opinions of others affect my test ordering], it’s if they weren’t involved in the procedure, I wouldn’t care, because I think that my practice is within guidelines, but if they were going to delay or cancel the procedure and become a barrier to us, then it would substantially influence me.” (S1)	2
		“This is a difficult part, because another physician has indicated that they should get it [the test] done. If I disagree and go on without the test, and then there’s a bad outcome—which is again, still unlikely, then it opens yourself up to legal ramifications.” (A6)	

Quotes are edited only for grammar and conciseness, when necessary

*A* anesthesiologist participant, *I* internist participant, *N* nurse participant, *S* surgeon participant

*Knowledge* alone did not drive low-value preoperative testing. Most participants were aware of preoperative testing guidelines (national, provincial, and/or institutional) and that these guidelines recommended against ordering ECGs and CXRs for patients undergoing low-risk surgeries based on peer-reviewed evidence. Even while reporting that these guidelines were helpful, most participants simultaneously expressed distrust of guideline quality and the supporting evidence. Participants felt that the role of guidelines was to guide clinicians rather than police decision-making, were only sometimes evidence-based, and included high- and low-quality evidence (knowledge).

Most clinicians felt that they did not need to order ECGs or CXRs to do their job and, while half of participants felt it was their responsibility to review these tests when they were available, the other half felt that it was not their responsibility to review tests that they did not order. Interestingly, while clinicians considered it part of their job to avoid ordering unnecessary tests, some internists reported that surgeon referrals often prompted them to order tests to justify the consultation or to order tests based on the belief that if the patient had been referred to them, there must be a reason (*social/professional role and identity, social influences*). The majority of clinicians also reported not needing to routinely order ECGs or CXRs; however, this intention contained a number of caveats where participants justified ordering tests based on age, medical condition, clinical necessity, and type of practice (i.e., oncology vs. non-oncology) (*motivation and goals*).

Lack of role clarity about which healthcare professional should order preoperative investigations and which healthcare professional should act on the results of these investigations was a central theme. For example, anesthesiologist and surgeon participants indicated that they should be responsible for ordering preoperative investigations, while internist participants had mixed opinions about whether they should or should not be responsible for ordering tests. Interestingly, while some respondents believed that other specialists were ordering preoperative testing when they should not be, most clinicals felt that the ability to order tests should not be restricted either (social/professional role and identity, social influences, environmental context and resources).

Most clinicians expressed that, with a good history and physical exam, they felt confident to proceed without testing, although some noted that this depended on the patient (beliefs about capabilities). Most clinicians reported that it is easy, and sometimes too easy, to order tests because the only thing they had to do was to ‘tick a box’ (beliefs about capabilities, environmental context and resources).

While a majority of participants felt that it was easy to not order low-value tests, many mentioned that cancelling ECGs or CXRs ordered by others was not easy (belief about capabilities). Some clinicians reported that they could not or would not cancel another physician’s order because this could be seen as an overreach into the other physician’s scope of practice which could potentially lead to legal issues or harm their relationships with colleagues and patient families if there was an adverse outcome due to not ordering tests (beliefs about capabilities, beliefs about consequences, social influences). Additional reasons for the difficulty to not order or cancel tests included the fact that tests were ordered automatically by the electronic health system/protocol, by nurses, or by the surgeon, and completed by the time they were seen by anesthesia or internal medicine to increase clinic efficiency and avoid increasing wait times (beliefs about capabilities, environmental context and resources).

There was variability in participant views on the consequences of not ordering preoperative low-value tests. The majority agreed that not ordering tests would save time, money, resources, reduce inconvenience and harm to patients, and decrease the chances of incidental findings. Despite this, participants reported that they may still order these tests to prevent surgery cancellations by an anesthesia colleague who preferred to have these tests available on the day of the procedure (beliefs about consequences, social influences). Conversely, some clinicians stated that they order tests to prevent problems during surgery and expressed concern over potentially missing something that could affect the patient for which they would be responsible (beliefs about consequences).

### Domains not found to be relevant

*Skills, memory, attention and decision processes, emotion*, and *behavioural regulation* were not identified as relevant domains (Table 3). All clinicians reported that anesthesiologists, nurses, internists, and residents should have experience and training to complete the patient history and physical exam and order appropriate preoperative tests. Most clinicians reported that the decision to order tests was easy, not automatic, and based on patients ‘ history, physical exam, medical conditions, and type of procedure (memory, attention, and decision processes). Most participants also expressed that their own emotions do not influence their test ordering but did worry about both unnecessary testing and potential adverse outcomes when tests are not ordered. Finally, behavioural regulation was not considered a relevant domain as participants did not currently have any strategies to reduce unnecessary testing and their suggestions for changing practice were already represented in the above-described domains as ways to address barriers and enablers.

**Table 3:** Summary of belief statements and sample quotes from anesthesiologists, internists, surgeons, and nurse assigned to the theoretical domains identified as not relevant

Domains	Specific belief	Sample quote	Frequency out of 16
Skills	Experience improves clinical skills/competence to perform preoperative assessments/order tests appropriately	“Because of years experience and also understanding the statistics around low-risk surgery that most patients are very low risk for low-risk surgery unless they have some major complications. […] So, understanding that through the experience, plus understanding the evidence around it.” (I2)	10
Memory, attention, and decision processes	The decision to order/not order tests is based on patients’ history, physical exam, and medical condition	“I guess the main thing that is, as I said, the main thing that this finds, if I’m ordering a test or not is the history and physical exam and if there is a new symptom or physical exam finding that wasn’t there before, that’s what defines if I’m ordering a test or not in the setting of a low-risk procedure.” (A2)	14
Emotions	My emotions do not influence whether or not I order ECGs/CXRs	“No. None. [managing a patient without ordering ECGs or chest x-rays evokes worry or concern]” (S1)	15
Behavioral regulation	Having clear decision tools/algorithms to streamline the process could reduce/reduces unnecessary testing	“…Strictly adhering to the guidelines, it could be something that could be done on a computer, maybe just obviously I think you would want to have probably a person just kind of reviewing maybe for each patient to make sure that what the computer suggested actually is appropriate. […] That would be very guideline driven and doesn’t need to be done by a physician. It could be done almost by a computer if you think about it. I guess in a perfect world, I think we as a system would benefit by being a bit more rigorous and structured in application of the guidelines through use of algorithms.” (I2)	7

## Discussion

This qualitative analysis of interviews with preoperative clinicians used the TDF (Michie et al. 2005) to understand the factors that influence the ordering of low-value preoperative tests in patients undergoing low-risk surgeries. The main drivers of low-value test ordering in our setting were both system- and team-level; specifically, a lack of role clarity around preoperative test ordering among preoperative clinicians, perceived interprofessional expectations, and a current process where tests are ordered before patients are seen by anesthesia or internal medicine to maximize clinic efficiency (social/professional role and identity, environmental context and resources). Our data highlights that low-value testing occurs partly because preoperative medicine is a complex process with multiple providers from different disciplines working in a variety of inpatient and outpatient settings in the absence of a structure that facilitates crosstalk within and across disciplines. Interventions to reduce low-value test ordering should address these factors.

These facilitators of low-value preoperative test ordering in Alberta were similar to those reported in Ontario (Patey et al. 2012). In both provinces, the lack of role clarity among the different specialists involved in the preoperative process contributed to low-value test ordering. In both settings, participants indicated that they ordered low-value tests to prevent surgical delays or day-of cancellations, based on the perception that their colleagues would be expecting these tests (beliefs about consequences, social influences). Similarly, the preoperative process in both provinces drives higher low-value test ordering by automatically sending patients for most tests before assessment by anesthesia and/or internal medicine. Similar findings were found in recent research from Saskatchewan (Shahid et al. 2021). However, unlike Ontario and Saskatchewan, clinicians in Alberta reported less trust in the quality of the evidence and guidelines that recommend against low-value tests before surgery and believed that individual patient factors justified higher rates of low-value testing (knowledge and motivation and goals).

The number of healthcare providers and the complexity of the preoperative process in Alberta contributed to many of the drivers of low-value test ordering in this setting. In Alberta, there are a greater number of specialists involved in the preoperative assessment compared to other Canadian jurisdictions, and any clinician involved in the preoperative process, including surgeons, anesthesiologists, nurses, and internists, could order low-value tests based on their own judgement. Due to the number of providers involved in preoperative test ordering, respondents described a dilution of responsibility for low-value test ordering, with many stating that these tests were either ordered by other clinicians or completed by the time patients were seen at the pre-admission clinic. Additionally, participants reported that most institutions had preoperative testing algorithms which were used by surgeons and/or nurses, rather than the anesthesiologists or internists. Interestingly, some participants reported that the algorithms for preoperative testing are created by anesthesiologists, and therefore, other specialists may believe that they are following the instructions written by anesthesiologists. In addition, nurses’ ability to order preoperative tests varied between settings both officially, based on a hospital’s institutional policies and unofficially, based on local practice. Furthermore, while most participants indicated that it was easy to not order tests, participants also reported that it was difficult to cancel tests ordered by other providers even when they felt that the tests were not indicated. This is consistent with previous research, which found that an important driver of low-value testing was anesthesiologist’s reluctance to cancel tests ordered by a surgeon prior to the preoperative assessment (Brown and Brown 2011). The ease of not ordering tests and difficulty of cancelling already ordered tests is consistent with the human tendency to choose the path of least resistance (Keijzers et al. 2018a). Altogether, this complex system favors low-value testing, as it only takes one of the many preoperative care providers to order an unnecessary test whereas all the preoperative care providers have to not order any unnecessary test to avoid low-value preoperative testing. For this reason, our results suggest that an intervention targeting only one group of providers or targeting individuals without addressing systems may be unlikely to reduce low-value test ordering.

These data suggest defining interdisciplinary roles for all providers involved in preoperative assessment is necessary to reduce low-value preoperative testing across healthcare systems. Role clarification, referring to “understand[ing] their own role and the roles of those in other professions, and use this knowledge appropriately to establish and achieve patient/client/family and community goals”, is a core competency in interprofessional collaboration (Canadian Interprofessional Health Collaborative 2010; Suter et al. 2009) that can reduce cost of care, outpatient visits, and clinical error rates while improving clinical outcomes and increasing patient satisfaction (Health Professions Networks Nursing & Midwifery for Human Resources of Health (2010). However, our study and others have shown that team members do not always acknowledge, understand, or respect each other’s roles (Kvarnström 2009; Larkin and Callaghan 2005). Role clarification has a positive impact of collaborative practice in improving access and coordination of health services, appropriate use of specialist clinical resources, and patient care and safety (Health Professions Networks Nursing & Midwifery for Human Resources of Health (2010); NHS Modernisation Agency 2004). Fisher found a 55% reduction in test ordering after the introduction of a clinical pathway that restricted preoperative test ordering to the anesthesiologist with an 88% reduction in day-of-surgery cancellations and 59% decrease in hospital costs with no increase in adverse patient outcomes (Fischer 1996). Role clarification can occur formally, through institutional policy or directives, or informally through discussions, interprofessional rounds, or agreements. Preoperative clinicians should decide who will determine which tests are required before surgery and plus clarification of professional expectations for what tests are or are not needed for surgery.

Further, our findings can guide design and selection of additional intervention components to reduce low-value preoperative testing. For example, interventions may need to address the ease of ordering and the speed at which tests can be performed (beliefs about capabilities), as clinicians currently only have to ‘tick a box’ to obtain tests. This supports previous research which suggests that redesigning the requisition forms (Emerson and Emerson 2001; Zaat et al. 1992; Mathura et al. 2021), limiting the test menu based on the physician’s specialty (Calderon-Margalit et al. 2005), or restricting how many tests physicians can order within a time period (i.e., every 8 h) (Neilson et al. 2004), are some of the strategies that reduce the number and frequency of tests ordered. In addition, institutional policies or processes that lead to preoperative tests being automatically ordered for all patients should be carefully reviewed and de-implemented in a thoughtful way. The NHS found that stratifying preoperative patients by their estimated surgical risk and creating evidence-based pathways for low-risk patients reduced low-value care while improving efficiency (Monitor 2015a, b, c). The implementation of this strategy required establishing standardised pathways and protocols created collaboratively by anesthesiologists, surgeons, and nurses, which detailed the roles and responsibilities of all the groups and guided the risk assessment (Helping and providers improve productivity in elective care. London. 2015). The creation of policies which clarify roles and adds barriers to low-value testing would also have a positive effect to counter physicians’ ‘intervention bias’ (Foy and Filippone 2013) and promote clinical inertia or “actively doing nothing as a positive response” (Keijzers et al. 2018a, b).

This study has several limitations. First, the setting was a single health system, and these results may not be transferable to different contexts. Organizations should undertake a barriers and enablers study in their own setting to understand what factors influence preoperative test ordering. Second, the results may not be representative of everyone’s views nor be considered as objective influences in the test ordering behaviour. For example, three of the four surgeon participants had an oncology practice, and their patients may not include patients undergoing low-risk surgeries. Interviewing surgeons in a non-oncological practice might provide additional insight to why surgeons order preoperative tests in patients undergoing low-risk surgeries.

## Conclusion

This study adds to growing evidence that guidelines or ‘education only’ interventions to reduce low-value preoperative testing do not address the drivers of ongoing low-value test ordering. Investigators and administrators aiming to reduce low-value test ordering can leverage implementation science expertise to understand their local context and drivers of low-value testing in their settings. They should consider role clarification of all clinicians who order preoperative tests and any directives that drive preoperative testing in their interventions. De-implementation strategies in Alberta need to encompass changes at an institutional- and team-level. By using theory-driven approach with the TDF, potential interventions linked to the theoretical domains, could be more effective at changing behaviour, and thus reduce unnecessary preoperative testing in patients undergoing low-risk surgeries (Grol et al. 2013; Davies et al. 2010).

## Supplementary Information

**Additional file 1.** Semi-structured Interview Guide (Pre-Operative Testing). Questions asked during the interviews and the definition of ‘low-risk surgery’.

## Data Availability

The datasets supporting the conclusions of this article are included within the article and its additional files.

## Abbreviations

TDF: Theoretical domains framework
RCT: Randomized controlled trial
ECGs: Electrocardiographs
CXRs: Chest X-rays
COREQ: Consolidated criteria for Reporting Qualitative Research guidelines
A#: Anesthesiologist
I#: Internist
N#: Nurse
S#: Surgeon
