# Supplementary material for: Beyond guideline knowledge: a theory-based qualitative study of low-value preoperative testing
Source: Perioper Med (Lond). 2023 Mar 2;12:3. doi: 10.1186/s13741-023-00292-5 (PMC9979452; doi:10.1186/s13741-023-00292-5)
Supplement: Supplementary file 1 — Additional file 1. Semi-structured Interview Guide (Pre-Operative Testing). Questions asked during the interviews and the definition of ‘low-risk surgery’. [file 13741_2023_292_MOESM1_ESM.docx]

**Additional File #1: Interview guide**

**Semi-structured Interview Guide (Pre-Operative Testing)**

*Introduction: I will turn the digital recorder on now. The general aim of the interview is to help us understand how you manage your pre-operative evaluations for low-risk procedures. We want to know what influences you when you are faced with the decision to order certain tests or not. There are no right or wrong answers here; we are trying to understand how different clinicians approach this issue, so please answer frankly.*

*I’d like to start with some basic questions about your practice:*

1. What type of setting do you practice in? (community vs. academic)

2. How many years have you practiced?

3. Do you practice or work closely with residents? How often (percentage of time)?

4. Could you walk me through your pre-op evaluation “process” for a patient undergoing a low-risk surgery? (Nature of behaviour)

5. As an anesthesiologist/surgeon/internist/nurse, how often do you come across patients undergoing low-risk surgeries? Percentage (Nature of behaviour)

*Thank you.*

*For the rest of the interview, I have some slightly more specific questions about what influences your decision regarding pre-operative testing for patients undergoing low-risk surgeries. Some questions may seem repetitive, but please bear with me as I need to ask all the questions for consistency. When this happens, you can tell me, “I already answered this” or add additional thoughts you may have about the topic.*

*Are you ready to get started?*

*So, I’d like to set the scene: I’m asking about “your pre-op evaluations for patients undergoing low-risk surgeries” – I’d like you to think about that for a moment…*

*When I say low-risk surgeries, I’m thinking about those patients having endoscopy, ophthalmology, or other low-risk procedures such as knee arthroscopy or urological procedures* *where patients are expected to be discharged on the same day* *and where only the principal intervention code (in the DAD) or first-listed intervention (in NACRS) was used to identify the low-risk procedure. Does that seem reasonable for low-risk/ambulatory?*

*Also, when I mention pre-op tests, I’d like you to focus on CXRs and ECGs.*

Knowledge

(What do they know and how does that influence what they do?)

6. Are you aware of any guidelines or recommendations (national, provincial or institutional) about pre-operative testing? (**If yes:** what do they say? / are they the only ones you know about? **If no:** Does your hospital have any policies about pre-op for low-risk surgeries?)

7. What are your thoughts about pre-op testing guidelines in general? (**Prompt:** Do you think there’s room for professional autonomy?)

8. Do you believe it to be evidence-based? What is your interpretation of the evidence?

Skills

(What do they know about how they should be doing something & how does that influence whether they do it or not?)

9. How much expertise or experience do you think one needs to have to perform a pre-op evaluation effectively? (**Prompt:** What about ordering a test?)

Nature of Behaviour

(What do you do and is that diﬀerent from what you should do?)

10. Do you routinely order CXRs and ECGs for patients having low-risk surgeries??

Memory, Attention and Decision Processes

(How does their forgetfulness or remembering to do it inﬂuence whether or not they actually do it? How does their ability to focus on the behaviour inﬂuence whether or not they do it? How do the decisions they make about the behaviour inﬂuence whether they do it or not?)

11. What thought processes might guide your decision to order pre-op tests for a patient having a low-risk surgery? (**Prompt:** “What goes through your mind?”)

12. Is managing a patient without ordering ECGs and CXRs an automatic part of your job, or is it something you take time to think about with patients having low-risk surgeries? (**Prompt:** routine, automatic)

13. Is it typically an easy or difficult decision to make? (**Prompt:** Weigh pros and cons etc.)

Social/Professional role & identity

(How does who they are as a HCP inﬂuence whether they do something or not?)

14. Is a review of a CXR or ECG an expected part of your pre-op check?

15. If you are doing a pre-op evaluation and you don’t order an ECG or CXR, do you think you’re doing your job? (**Prompt:** are these two tests fundamental to you doing your job (part of an effective evaluation?)) (Are they fundamental in your role? Are they always done because they’re part of being a surgeon/anesthesiologist/internist/nurse?)

(a) Who do you think should be responsible for ordering ECGs and CXRs for low-risk surgeries (which group of physicians)? Why? Do you see these tests as primarily for anesthesia management in low-risk surgeries, or are there additional reasons for ordering these tests? Why?

16. Is there anything in your professional role that influences your decision to order or not order certain tests for a patient having a low-risk surgery? (**Prompt:** professional training, a protocol, an order set, other technologies)

Environmental Context & Resources

(What are the things in their environment that inﬂuence what they do and how do they inﬂuence? (not just physical stuﬀ, but access to other professionals))

17. What aspects of your clinical environment (physical vs. resource factors) influence whether or not you order tests for a pre-op evaluation for a patient having a low-risk surgery?

18. Are there any competing tasks or time constraints that might influence whether or not you order tests for a pre-op evaluation for a patient having a low-risk surgery?

(a) What would help you overcome these problems/difficulties? (**Prompt:** skills training in the medical curriculum, communication techniques, formal training programs, CME, educational material online or by mail)

Beliefs about Capabilities

(Do they think they can do what they should do and how does that inﬂuence whether they do it or not?)

19. How easy or difficult is it for you personally to order tests during a pre-op evaluation? Why or why not?

20. How easy or difficult is it for you personally to cancel or order no tests at all? Why or why not?

21. Are you confident that you are able to perform a pre-op evaluation for a low-risk surgery without pre-op tests? How have you gained that confidence?

22. Would you be comfortable proceeding without testing? Why or Why not?

Social Influences

(What do other’s think of what they do? Who are they and how does that inﬂuence what they do?)

23. Would any other team members influence whether or not you order certain tests for a pre-op evaluation for a patient having a low-risk surgery? (**Prompt:** who else; other clinicians; medical staff including nurses and residents/fellows; relatives; surgeons test orders)

24. How might the views/opinions of others affect your ordering certain tests for a pre-op evaluation for a patient having a low-risk surgery?

25. Do your colleagues generally agree with you on this issue?

26. Do patient emotions ever influence whether or not you order certain tests for a pre-op evaluation for a patient having a low-risk surgery? Explain.

Emotion

(How do they feel about what they do and do those feelings inﬂuence what they do?)

27. Does managing a patient without ordering ECGs and CXRs in a pre-op evaluation for a low-risk surgery evoke worry or concern in you?

Beliefs about Consequences

(What are the good and bad things that can happen from what they do, and how does that inﬂuence whether they’ll do it in the future? How have their experiences (good and bad) of doing it in the past inﬂuence whether or not they do it?)

28. Do you believe the costs of pre-op testing are worth the benefit in low-risk surgeries? Why or why not?

29. What do you think will happen if you don’t order ECGs or CXRs during your pre-op evaluation, both positive and negative? (**Prompt:** to patients, to colleagues, yourself, short and long term)

Motivation and Goals

(How important is what they do & does that inﬂuence whether or not they do it? What standards are they trying to reach, how does that inﬂuence whether or not they do it?)

30. In your pre-op evaluation of a patient having a low-risk surgery, do you plan to order ECGs and CXRs routinely? Is it something you feel you need to do?

31. How important is it to you to perform pre-op tests in your pre-op evaluation of a patient having a low-risk surgery?

32. What would be an incentive for you to reduce the number of pre-op tests you order when evaluating patients for low-risk surgeries? (**Prompt:** goals within yourself? external?)

Behavioural Regulation

(What do they think would help them/what strategies have helped them do what you should do? What strategies are already in place to help them do what they should do?)

33. When faced with a patient preparing for a low-risk surgery, would managing without ordering ECGs and CXRs be something you would usually do?

34. What would you, personally, have to do to decrease the number of pre-op tests you order for a patient having low-risk surgery?

🡪 **Follow-up:** In an ideal world, where anything is possible, what would you see as the best process for ordering pre-op tests for a patient having a low-risk surgery?

35. If you wanted to implement changes in your own practice (individual/team setting/practice setting) to discourage pre-op testing for low-risk surgeries, what would be some ways to do this? (**Prompt:** role of protocol or guidelines)

That’s all the questions I have for you; has anything occurred to you about this topic that we haven’t asked about?

Thank you!

**Turn digital recorder off and snowball sampling:**

*“I have turned the digital recorder off now, and would like to inquire whether you would be willing to identify a few other colleagues who may have differing opinions than yours, and who may be interested in speaking with us about their perspectives?”*

**If they say ‘yes’ 🡪** record relevant names for follow-up recruitment. “We would like to provide you with a $100 gift card in appreciation for your time and insights.”

**If they say ‘no’ 🡪** *“Thank you very much for participating. That concludes our interview. We would like to provide you with a $100 gift card in appreciation for your time and insights.”*
